# Adult Urethral Stricture Disease after Childhood Hypospadias Repair

**DOI:** 10.1155/2008/150315

**Published:** 2008-11-04

**Authors:** Shou-Hung Tang, Clarisa C. Hammer, Leo Doumanian, Richard A. Santucci

**Affiliations:** ^1^Department of Urology, Tri-Service General Hospital, Neihu, Taipei 114, Taiwan; ^2^Department of Surgery, St. John Oakland Hospital, Madison Heights, MI 48071, USA; ^3^Center for Urologic Reconstruction, Detroit Receiving Hospital, University Health Center, MI 48201, USA

## Abstract

*Background*. Adult patients with urethral stricture after childhood hypospadias surgeries are infrequently discussed in the literature. We report our experience in treating such patients. *Materials and Methods*. A retrospective chart review was performed. From 2002 through 2007, nine consecutive adult patients who had current urethral stricture and had undergone childhood hypospadias surgeries were included. All adult urethral strictures were managed by a single surgeon. *Results*. Mean patient age was 38.9 years old. The lag time of urethral stricture presentation ranged from 25 to 57 years after primary hypospadias surgery, with an average of 36 years. Stricture length ranged from 1 to 17 cm (mean: 10.3 cm). Open graft-based urethroplasties were performed in 4/9 cases. Salvage perineal urethrostomy was performed in 2/9 cases. Another 3 cases chose to undergo repeat urethrotomy or dilatations—none of these patients was cured by such treatment. Complications included one urethrostomy stenosis and one urinary tract infection. *Conclusion*. Urethral stricture may occur decades after initial hypospadias surgery. It can be the most severe form of anterior urethral stricture, and may eventually require salvage treatment such as a perineal urethrostomy. Patients undergoing hypospadias surgery should receive lifelong follow-up protocol to detect latent urethral strictures.

## 1. INTRODUCTION

Hypospadias is a common congenital abnormality occurring in 1/300 live births, and is the most common congenital penile anomaly [1, 2]. Numerous surgical techniques have been developed to correct this anomaly. However, no single method is considered the standard of care, and they all share the common complications of occasional urethrocutaneous fistula and urethral strictures. The incidence of urethral stricture after hypospadias surgery in pediatric population is reported, and occurs in about 6.5% after short followup [3]. On the other hand, there are few reports dealing with urethral strictures in adults after they had hypospadias surgery in childhood. In the current series, we described our experiences in 9 such cases, and review their particular characteristics and suggested treatments.

## 2. MATERIALS AND METHODS

A retrospective chart review was performed from 2002 through 2007. Nine consecutive adult patients who had current urethral strictures and had undergone childhood hypospadias surgeries were included in this study. All adult urethral strictures were managed by a single surgeon (RAS). The strictures in these patients were all symptomatic and were documented by retrograde urethrography (RUG). Information regarding hypospadias repairs, previous urethral manipulations, presenting symptoms, stricture length, definite treatment, and short-term outcomes were obtained from medical records. In cases undergoing perineal urethrostomy, we suggested the “side-to-side” technique, which comprised longitudinal urethrotomy and everting the mucosal and submucosal layers of the urethra to the incised skin. Graft-based urethroplasty using buccal mucosal graft, in one-stage or two-stage repairs, was the preferred choice of formal reconstruction.

## 3. RESULTS

Mean patient age was 38.6 years old, and mean followup period was 1.9 years. All patients had their primary hypospadias surgeries between 1 and 12 years old. The lag time of the adult urethral stricture presentation ranged from 25 to 57 years, with an average of 36 years between hypospadias surgery and presentation to our clinic with urethral stricture.

**Figure 1:**
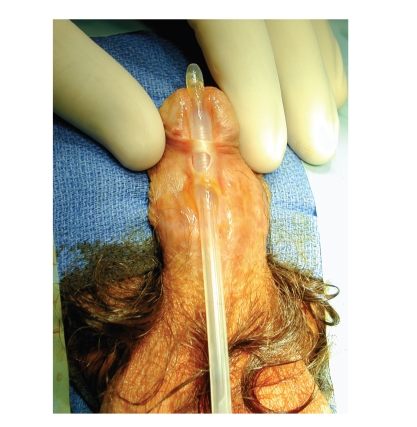
Typical complex urethral stricture after childhood hypospadias repair, with a distal penile location and complicating fistulae.

Four of the 9 patients (44%) presented with acute urinary retention, and one of these patients developed acute renal failure due to prolonged urinary retention, before stricture was diagnosed. The other associated complications included fistula in one case. Only three of the 9 patients (33%) suffered from lower urinary tract symptoms (LUTS) including decreased voiding stream, spraying, dribbling, and nocturia. In one case, urethral stricture was discovered when we evaluated unresolved urethrocutaneous fistulas (see Figure 1). Before transferred to our institute, 5/9 patients had undergone endoscopic treatment for strictures, and 2/9 had failed open urethroplasties.

**Figure 2:**
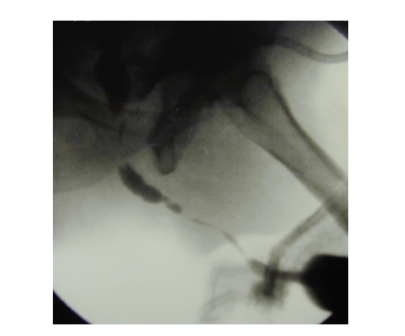
Typical retrograde urethrogram appearance of adult stricture after hypospadias repair showing a long stricture sparing only the bulbar urethra.

Penile urethra was involved in all cases, and bulbar urethra was involved in 5/9. The stricture length ranged from 1 to 17 cm (mean, 10.3 cm) (see Figure 2, Table 1)

**Figure 3:**
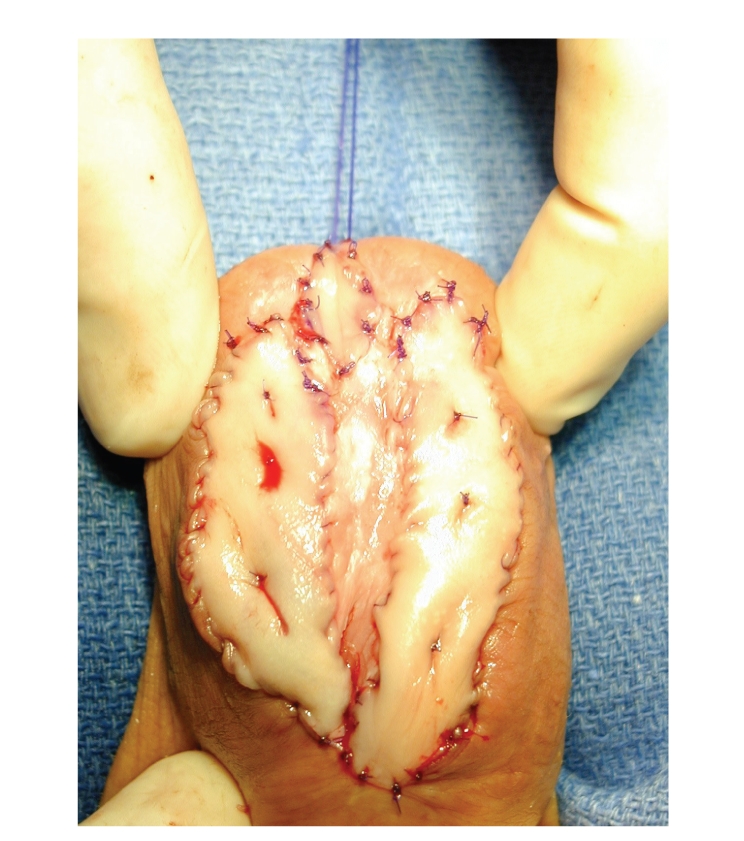
First-stage urethroplasty with buccal grafts.

**Figure 4:**
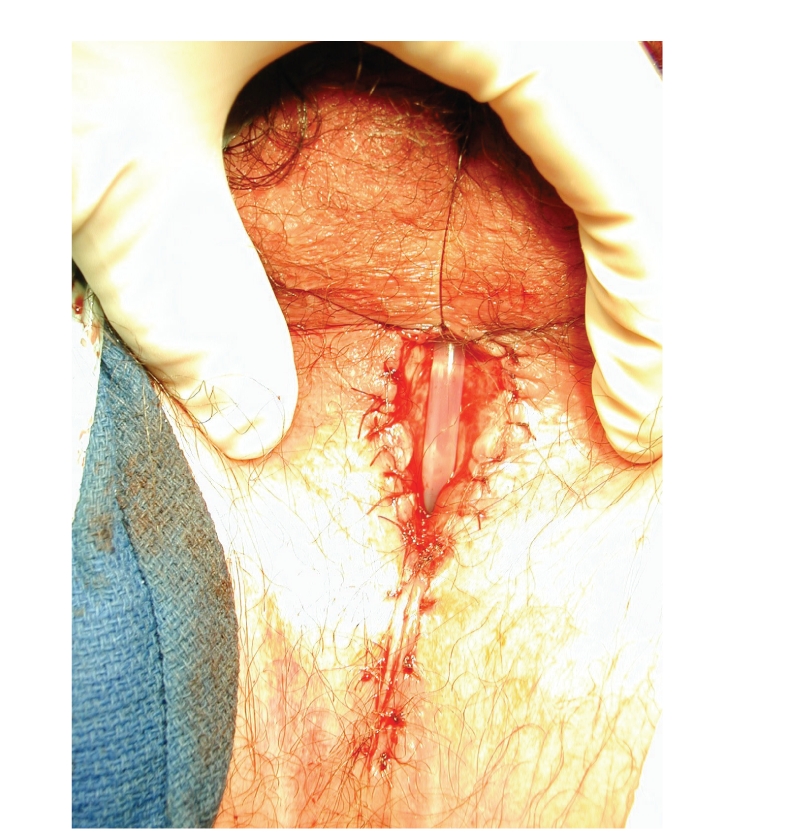
Perineal urethrostomy, performed using our suggested “side-to-side” technique.

Open urethroplasties with buccal mucosal grafts were performed in 4/9 cases: two with single-stage repair and two with 2-stage repair with buccal grafts (see Figure 3). Salvage perineal urethrostomy was performed in 2/9 cases, usually in patients who did not wish complex definitive urethral surgery (see Figure 4). The perineal urethrostomy was planned to be permanent. Another 3 cases chose to receive repeated endoscopic treatments (direct visual internal urethrotomy (DVIU) and/or dilatations) although perineal urethrostomy or urethroplasty had been offered.

None was cured by DVIU/dilatations. Four patients receiving open urethroplasty with buccal grafts were free of stricture recurrence. Stenosis of urethrostomy developed in one case and was successfully managed by a V-Y plastic technique.

## 4. DISCUSSION

There is little literature mentioning adult urethral stricture in hypospadias patients. Barbagli et al. published a series of 60 adults with previously failed hypospadias repair [4], including 34 cases that underwent treatment for urethral strictures. Their overall successful rate was 75% (83% for one-stage repair, 68% for multistage repairs). It was evident that those who needed multistage repair plans were at higher risk of failure because they had more severe strictures and extremely poor quality native tissue than those on whom the surgeons would consider risking a single-stage repair.

### 4.1. How are these hypospadias stricture patients different?

Adult stricture patients with previous hypospadias repair differ from a usual population of stricture patients. First, they sometimes had no voiding complaints even when their strictures were severe. Second, they had complicating problems seldom seen in other stricture patients, including complete renal failure and urethral fistula. Third, they have a poor quality of tissue which requires more complex repairs such as first-stage Johanson operations, with buccal grafts placed in the first stage, followed by second-stage closure later. The associated complications do represent a factor influencing the surgical strategy. However, major determinants were the stricture length, availability of healthy tissue, as well as surgeon's own preference. In patients with long stricture and prominent scarring, we suggested staged repairs if formal reconstruction was planned. Last, they often have such long and hopeless abnormal anterior urethras, that is, by both patient and surgeon, it is determined best to treat them expediently with simple perineal urethrostomy instead of formal repair. In this way, reliable egress of urine can be virtually guaranteed after a short 1-2 hour operation, an option chosen by 5/9 (56%) of our cases. Our experience here exemplified that heroic measures were not always justified to treat the severest urethral strictures, and that perineal urethrostomy can be a gratifying option.

### 4.2. Healing in adults and children

While differences in wound healing ability between children and adults are well described [5], little direct data is available on the relative behavior of adult and childhood tissue in the urologic arena. Adult hypospadias surgery has been reported, and may provide some insight into the pitfalls of complex reconstructive surgery in the adult. For example, in a series of adults who underwent adult hypospadias repair, redo operations had a worse outcome than primary cases [6]. They found that previous surgeries and poor tissue quality attributed to higher failure rates. There was a significant difference in terms of wound healing, infection, complication rates, and overall success in adults compared to children. Increased surgical difficulty and high failure rate after redo adult hypospadias surgery may be well applied to adult urethral strictures such as seen in our population.

### 4.3. Incidence of adult urethral stricture after childhood hypospadias repair

Urethral stricture is a known complication following hypospadias repair [7–9], but the true incidence is unknown. Some childhood hypospadias series do not follow the patients long enough to report any strictures, and when series do report strictures, they usually report them as acute events that occur while the patient is still in childhood, not later as adults. A series by Duel et al., for example, showed a stricture incidence as high as 6.5% (38 of 582) after pediatric hypospadias surgeries [3]. They demonstrated that strictures occurred after a (mean) interval of 27 months. 79% of these pediatric urethral strictures ultimately required open urethroplasty for correction, and they had a 78% overall successful rate.

The wide range of stricture length in our series was mainly affected by original type of hypospadias. Patients undergoing repair for scrotal hypospadias would have greater chance to have a subsequent longer urethral stricture. Unfortunately, the exact type of original hypospadias cannot be determined simply by gross appearance or by history in most cases.

### 4.4. How to treat the adult stricture patient

Repair of posthypospadias strictures in children has been widely discussed by pediatric urologists. Modern series now favored single-stage, two-stage buccal mucosal graft repair, or urethroplasties utilizing tunica vaginalis [10, 11]. We agree, and tend to offer two-stage buccal mucosal repairs such as described by Johanson in adults. We also acknowledge that some of these patients have such extensive disease, and little interest in a two-stage operation to fix the problem, and thus are most appropriately treated with a perineal urethrostomy. Perineal urethrostomy was offered as a second choice in addition to formal urethral repair. Comorbidity and previous failed urethroplasty were the major factors influencing patients who accepted perineal urethrostomy.

## 5. CONCLUSION

Urethral stricture can occur decades after initial hypospadias surgery. Patient often have few voiding complaints and can present with severe complications. The stricture can be very extensive and may require salvage treatment such as a perineal urethrostomy. Two-stage urethroplasties with buccal mucosal grafts can achieve good result when necessary. We suggest that patients undergoing hypospadias surgeries should receive lifetime followup to detect latent urethral strictures, and that research reports discussing stricture after hypospadias repair include very-long-term followup data to determine the exact incidence of this problem.

## Figures and Tables

**Table 1:** Characteristics of 9 adult urethral stricture patients with childhood hypospadias repair.

age	length (cm)	treatment	recurrence
25	1	Single-stage dorsal-onlay urethroplasty with buccal mucosal graft	no
26	6	Single-stage dorsal-onlay urethroplasty with buccal mucosal graft	no
39	4	Two-stage urethroplasty with buccal mucosal graft in first stage	no
55	5	Two-stage urethroplasty with buccal mucosal graft in first stage	no
26	17	DVIU/dilatations	yes
48	13	DVIU/dilatations	yes
37	15	DVIU/dilatations	yes
36	15	perineal urethrostomy	no
57	17	perineal urethrostomy	Yes, Y-V plasty
